# The methylation level of TFAP2A is a potential diagnostic biomarker for retinoblastoma: an analytical validation study

**DOI:** 10.7717/peerj.10830

**Published:** 2021-03-02

**Authors:** Qi Zeng, Sha Wang, Jia Tan, Lu Chen, Jinwei Wang

**Affiliations:** 1 Hunan Provincial People’s Hospital (The First-Affiliated Hospital of Hunan Normal University), Changsha, China; 2 Eye Center of Xiangya Hospital, Central South University, Changsha, China; 3 Hunan Key Laboratory of Ophthalmology, Changsha, China

**Keywords:** DNA methylation, Retinoblastoma, Cell free dna, Diagnostic biomarker, Liquid biopsy, ctDNA

## Abstract

Tumor-derived circulating tumor DNA (ctDNA) has demonstrated its excellent potential for cancer diagnosis by DNA methylome; therefore, this study aimed to identify the retinoblastoma (RB) specific methylated CpG loci as the RB diagnostic biomarkers and design a methylation specific assay to detect these biomarker from aqueous humor of RB patients. Through a genome-wide methylation profiling of tissue samples from patients with RB, normal retina and other retinal diseases, we shortlisted two CpG loci were only methylated in RB but not in normal retina or other retinal diseases. Both of these two CpG loci were located in the genome of TFAP2A. Through the screening, a primer and probe set for the two CpG loci were tested in fully methylated standards and RB tissues with a significant differentiation of RB. Our results of this assay tested in aqueous humor from RB revealed an accuracy of 92.7% for RB diagnosis. These results suggested our assay targeting the TFAP2A ctDNA methylation can be utilized for RB diagnosis and cancer monitoring.

## Introduction

Retinoblastoma (RB) is a primary cancer that develops in the eyes of children. Although there are multiple treatments, it is necessary to remove the eyeball or surgically remove the entire eye for advanced tumors (Fabian et al., 2018; Mendoza & Grossniklaus, 2016). The International Intraocular Retinoblastoma Classification (IIRC) scheme was developed to classify the intraocular RB. Currently, based on clinical classification, including tumor size, retinal detachment and tumor seeding (Linn Murphree, 2005), physicians can predict which eye will respond to treatment (and avoid removing the eyeball) if the patients are classified in IIRC group A, B or C. For advanced RB (group D and E), the predictive accuracy of eye salvage will drop down to below 50%. The diagnosis for RB mainly depends on the history, ophthalmic exam or radiological exams such as MRI and ultrasound. These tools are helpful in the differential diagnosis of RB from other retinal diseases in most cases, i.e., congenital cataract, prematurity retinopathy, vitreous haemorrhage (Berry et al., 2018) but there sometimes are dilemmas. Therefore, a new tool specific to RB is required now.

Epigenetic events are the strong candidate for early detection of disease because aberrant regulation of gene expression by DNA methylation is a well-characterized event in tumor biology and is extensively described for cancer diagnosis (Yokoi, Yamashita & Watanabe, 2017; Pan et al., 2018; Xie et al., 2018; Liang et al., 2019; Natale et al., 2019; Fece de la Cruz & Corcoran, 2018; Locke et al., 2019; Davis et al., 2020). In addition, the methylation patterns can be used for mapping the tissue of origin, which elevate the clinical application of DNA methylation in cancer diagnosis (Lehmann-Werman et al., 2016; Moss et al., 2018). To date, circulating cell free DNA (cfDNA) is the liquid biopsy analyte most extensively subjected to methylation analyses. Therefore, a few targeting abnormal cfDNA methylation level assays have demonstrated their clinical utility for cancer (early) diagnosis (Nawaz et al., 2014; Shen et al., 2018) and this fraction of cfDNA derived from cancers is defined as circulating tumor DNA (ctDNA). Of them, testing for the screening of early colorectal cancer is among the most frequent uses of ctDNA methylation based assay. Highly methylated patterns are observed in BMP3, NDRG4 and SEPT9 in stool or blood samples from colorectal cancer patients (Chen et al., 2019; Ahlquist et al., 2012; Tepus & Yau, 2020). The development of DNA methylation biomarkers is also emerging in other cancers, such as GSTP1 for prostate cancer (Zhao et al., 2018), SHOX2 and RASSF1A for lung cancer methylation (Zhang et al., 2017) and OTX1 for bladder cancer (Van Kessel et al., 2016). Such attempts to cancer diagnostic biomarkers highlighted the clinical usage of epigenetic biomarkers.

A large-scale cohort comparing the accuracy of ctDNA methylation assay and genetic alteration has uncovered the potential usage of pan-cancer cfDNA methylation assay (Taylor, 2020). Through the bioinformatic analyses of TCGA illumine 450k datasets including multiple cancers and normal tissues, a panel of cancer specific DNA methylation biomarkers can also suggest the tissue of origin. Due to the blood-retina barrier, ctDNA are enriched in aqueous humor (AH) rather than the peripheral blood. Previous study also demonstrated the feasibility of ctDNA isolation from small amount AH and ctDNA could act as a monitor biomarker in RB using copy number alteration (Berry et al., 2018). In this study, we identified a region of two CpG loci are RB specific biomarker via integrated bioinformatic analyses. A DNA methylation specific PCR assay targeting this region was designed and tested in RB tissue and aqueous humor samples and demonstrated a remarkable accuracy in clinical practice.

## Methods

### Data processing

The dataset containing RB, normal retina and other retinal diseases was retrieved from a public methylation data repository (GSE57362) by the R package “GEOquery”. We downloaded a public dataset (GSE57362) from the DNA methylation array repository. The whole datasets contained 256 retinal samples involving variety of retinal diseases. Since the aim of this study is to identify the specific hypermethylated CpG loci in RB, we removed embryonic retinal samples and samples from in vitro cell lines. The original dataset contained 445566 probes covering the genome-wide CpG loci (Berdasco et al., 2017). The annotation files from the package “IlluminaHumanMethylation450kanno.ilmn12.hg19” were used for CpG loci annotation. We removed the CpG probes located in sexual chromosomes. We also removed those probes with crossing-reactions afterwards (Chen et al., 2013). The beta values were utilized for data visualization while M values were used for the statistical analyses.

### DNA methylation marker identification

The bioinformatic analyses were performed according to the DNA methylation analysis workflow (https://www.bioconductor.org/packages/release/workflows/vignettes/methylationArrayAnalysis/inst/doc/methylationArrayAnalysis.html) with a little modification. All bioinformatic analyses were conducted in R 3.6.3 (https://cran.r-project.org/). Specifically, for the RB specific DNA methylation biomarker identification, we utilized multiple steps to screen the hypermethylated CpG loci in RB. First, we performed a linear model to identify the hypermethylated CpG loci in RB as compared with normal retina samples using the package “limma”. The CpG loci with log fold change larger than 2 and the adjusted *p* values less than 0.01 were considered as the RB specific biomarker candidates. Then, we filtered out these non-RB-specific CpG loci (of which average beta values were larger than 0.2 in diabetic retinopathy). Finally, two CpG loci met our selection criteria and were considered as the RB specific biomarkers. The genomic regions of these two loci flanking 100 bp up-and down-stream were the region of interest for methylation specific PCR design.

### Sample collection and bisulfite conversion

Institutional Review Board approval was obtained with written informed consent from the parents of participants. Samples were sequenced within 1 month of extraction. This study included patients diagnosed with RB from December 2018 to September 2019 who underwent surgical operation for removal of RB before routine clinical treatment. The aqueous humor (0.1 mL) or RB tissue samples of patients with RB were carried out and parental consent was obtained. Control samples included 3 cases of congenital glaucoma and 2 cases of pediatric cataract patients with 0.1 mL AH. There was no history of infectious or inflammatory before. REMARK guideline for reporting biomarkers was followed.

Tissue or AH samples were stored at -80 Celsius degree immediately after isolated from patients. For AH samples, we spanned these samples at the speed of 20, 000X g for 5 min to remove cell debris and apoptotic body. The genomic DNA from tissue or cfDNA from AH was isolated by QIAamp DNA Mini Kit (Qiagen) or QIAamp Circulating Nucleic Acid kit (Qiagen), respectively. DNA concentrations were assayed using Qubit HS (High-Sensitivity) kits (Thermo Fisher) and DNA was eluted in 50 uL. The EpiTect Fast Bisulfite Conversion kit (Qiagen) was used to bisulfite convert isolated tissue genomic DNA and cfDNA.

### Methylation specific PCR (MSP)

The methylation specific PCR primer pairs for TFAP2A were designed by MethPrimers (http://www.urogene.org/cgi-bin/methprimer2/MethPrimer.cgi) with the following parameters: Tm: 60; amplicon length: 90 bp. The primers for the internal control ACTB were adapted from a previous study (Warren et al., 2011). This ACTB region had no methylated CpGs and was utilized to monitor the input cfDNA quality after bisulfite conversion and total cfDNA amount in PCR reaction. The sequences of primers and probes were listed in Table S1.

Each PCR run included three no template control samples. The determination of methylated RFAP2A in genomic DNA or cfDNA was measured on triplicate input of 10 ng bisulfite converted DNA using methylation specific PCR (MSP). In brief, the real-time PCT assays targeted methylated CpG loci in the region of interest. DNA target amplification was performed for 50 cycles in an LightCycler 480 (Roche). Ct values were calculated using a 2nd derivative algorithm provided with the LightCycler 480 software. The MSP assay of TFAP2A was considered as “positive” if a total change in fluorescence intensity above background levels was measured within 50 PCR amplification cycles. To integrate this information into the final quantification of methylation levels of TFAP2A, we compared the 2 delta Ct of each methylation detection repeat with the average Ct of ACTB.

### Statistical analyses

Quantitative data were expressed as the median (interquartile range) of continuous (nonparametric) variables and the frequency (percentage) of categorical variables. For comparisons between groups, Student t test was used for continuous data, while Fisher’s exact test was used for categorical data. The 2 delta Ct values of individual genes to determine the performance of each individual marker (R statistical software, version 3.0.2; Reference 31), and perform receiver operating curve (ROC) analysis. The area under the curve (AUC) had a 95% confidence interval (CI). A *p* value less than 0.05 was considered as statistical significance.

## Results

### Characteristics of the patients

A total of 15 patients with RB and 5 patients with non-RB were included in this study. The AH samples were isolated from all participants with consents. Of them, additional five RB tissue samples were extracted for RB genomic DNA. The tissue and AH samples were obtained and stored in -80 Celsius degree before the surgical resection of RB. The demographic characteristics of the 15 RB patients were listed in the Table 1.

**Table 1:** The demographic characteristics of patients included in this study.

	**Patients**	**Tissue samples**	**Gender**	**Disease**	**Age**	**Literality**	**RB1 mutation**	**ctDNA conc.**	
									
	1		Female	RB	20 months	unilateral	Negative	0.23	
	2	obtained	Male	RB	7 months	bilateral	Positive	0.43	
	3		Male	RB	15 months	bilateral	Positive	0.76	
	4	obtained	Male	RB	2 months	bilateral	Positive	0.93	
	5		Female	RB	16 months	unilateral	Negative	0.19	
	6		Male	RB	6 months	bilateral	Negative	0.92	
	7	obtained	Female	RB	9 months	unilateral	Positive	5.39	
	8		Female	RB	16 months	unilateral	Negative	2.57	
	9	obtained	Female	RB	21 months	unilateral	Negative	0.77	
	10		Male	RB	21 months	unilateral	Negative	7.26	
	11		Male	RB	10 months	bilateral	Positive	10.2	
	12	obtained	Female	RB	5 months	unilateral	Negative	5.37	
	13		Female	RB	24 months	unilateral	Positive	29.4	
	14		Female	RB	13 months	bilateral	Positive	16.3	
	15		Male	RB	8 months	bilateral	Positive	0.86	
	16		Male	congenital glaucoma	17 months	NA	NA	0.12	
	17		Male	congenital glaucoma	20 months	NA	NA	0.14	
	18		Female	pediatric cataract	4 years	NA	NA	0.09	
	19		Female	pediatric cataract	5 years	NA	NA	0.17	
	20		Male	pediatric cataract	8 years	NA	NA	0.17	

The concentration of circulating DNA was determined for the samples from 15 RBs and 5 controls. The median concentration of cfDNA from AH of RB is 0.93 ng/uL (range: 0.19–29.4 ng/uL) while the median concentration of cfDNA from controls is 0.14 ng/uL (range: 0.09–0.17 ng/uL). The details of cfDNA concentration for each patient were listed in the Table 1.

### Hypermethylated loci in RB

**Figure 1:**
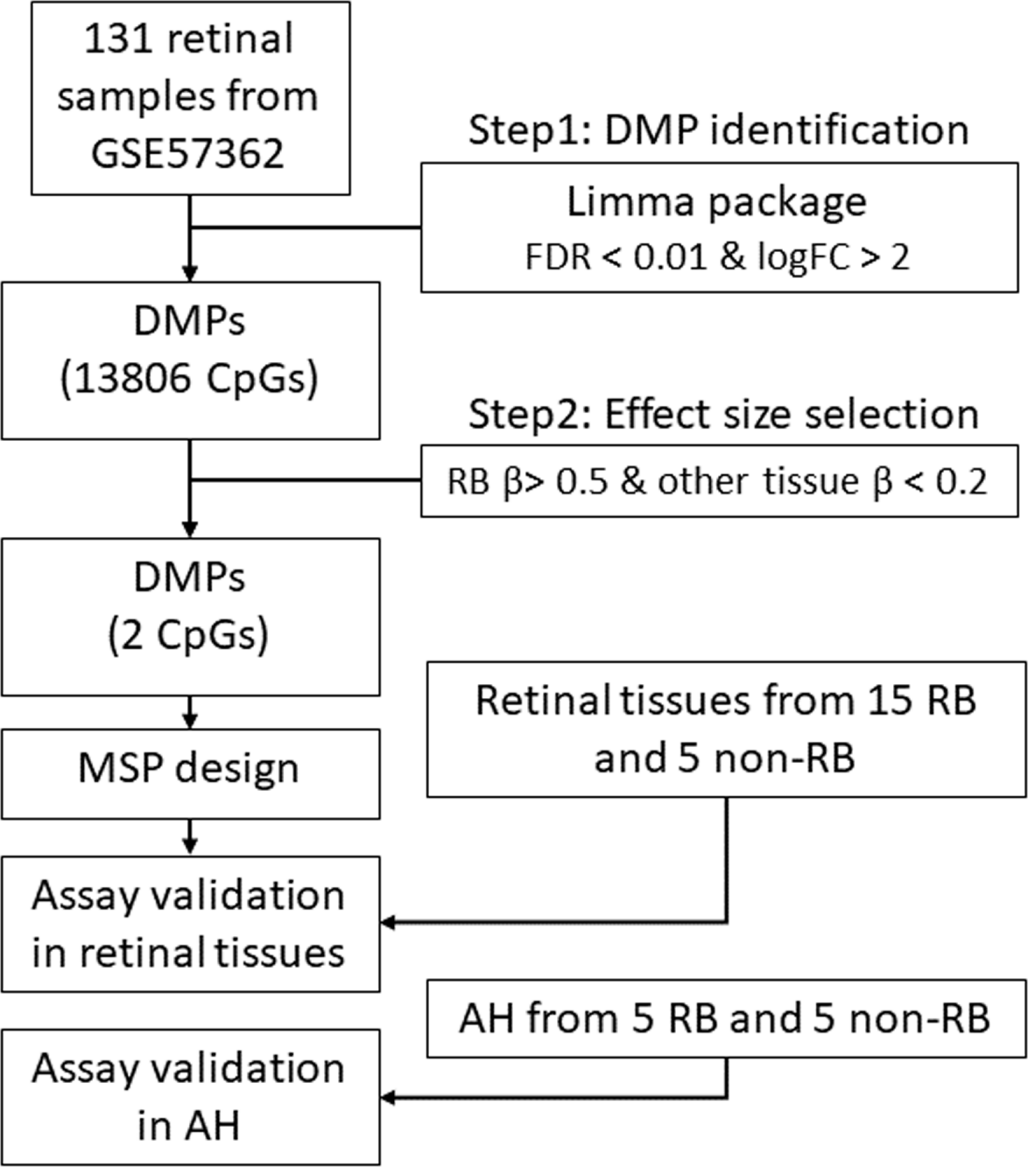
The workflow of this study. DMP, differential methylation probe; FDR, false discovery rate; FC, fold change; RB, retinoblastoma; MSP, methylation specific PCR.

**Figure 2:**
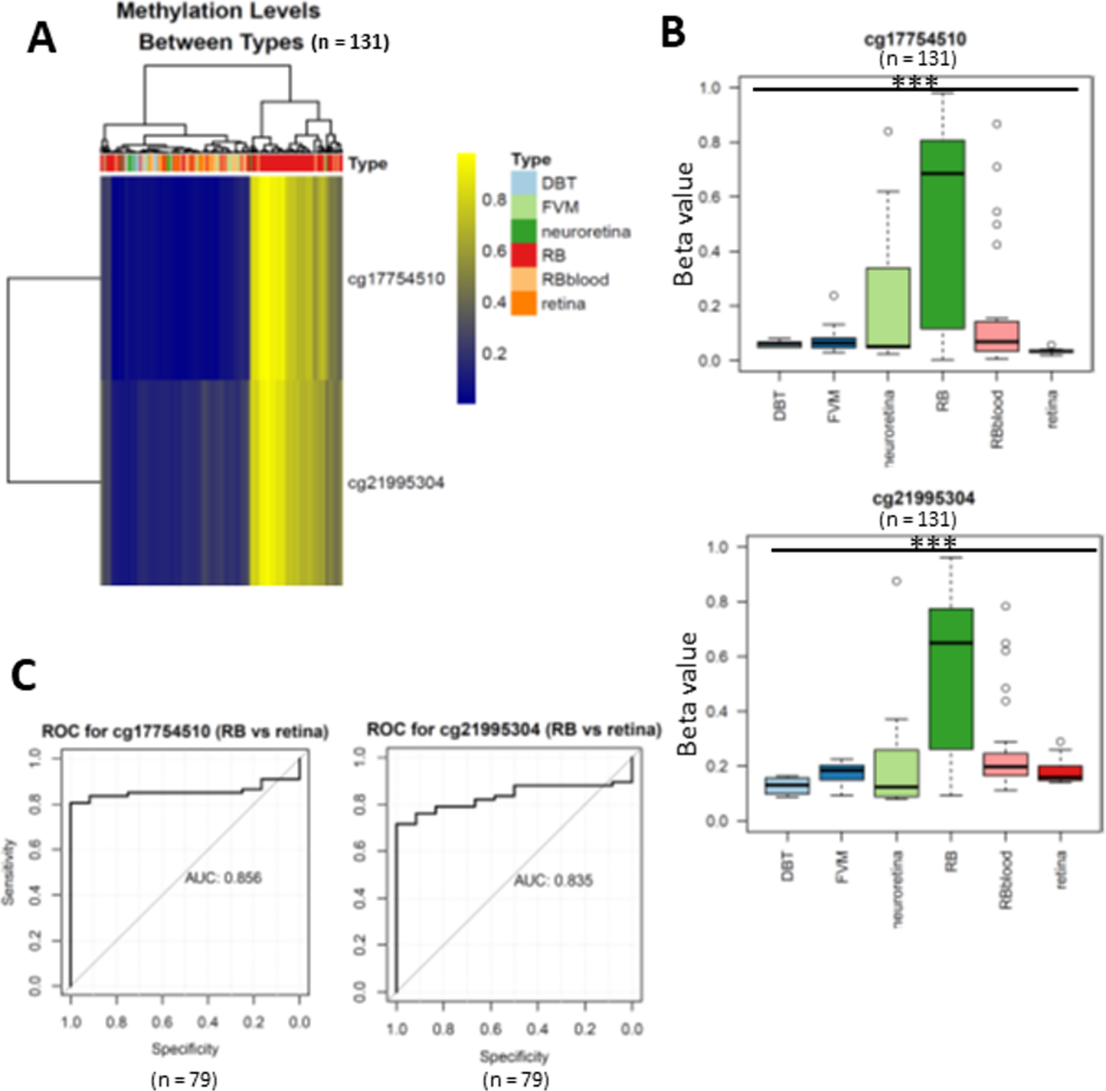
Differentially expressed methylated CpG loci in RB. (A) heatmap showing two CpG loci (cg17754510 and cg21995304) were hypermethylated in RB. (B) Boxplots showing the methylation levels of two CpG loci (cg17754510 and cg21995304) were significantly higher than normal retina and other retinal diseases. (C) the ROC showing the discriminative capacity of the two CpG loci between RB and normal retina.

To uncover the specific methylation markers for RB, we utilized a public dataset GSE57362 containing 67 RBs, 12 normal retina, 8 non-proliferative diabetic retinopathy (DBT), 8 neuroretina, 9 Fibrovascular membranes from diabetic retinopathy (FVM) and 27 peripheral blood samples from patients with RB. After downloading and pre-processing the dataset, we performed a differentially expressed methylation analysis on it by limma. As we aimed to identify the RB specific methylated markers, we only selected the probes that were significantly hypermethylated in RB as compared with normal retina. To further select the RB specific methylated markers, we only retained the probes of which the beta value larger than 0.5 in RB while less than 0.2 in DBT and FVM (Fig. 1). At last, two probes (cg17754510 and cg21995304) were remained via our filtration (Fig. 2A). We also observed a significant difference of beta value in these two CpG loci between RB and normal retina as well as other retinal diseases (Fig. 2B), suggesting these two CpG loci were potentially diagnostic biomarker for RB. To further validate the diagnostic utility of these two CpG loci, we performed a Receiver Operation Curve (ROC). The results from ROC demonstrated a remarkable separation of RB from normal retina and other retinal diseases where the Area Under the Curve (AUC) of cg17754510 and cg21995304 were 0.856 and 0.835, respectively (Fig. 1C). Interestingly, we found both two CpG loci were located in the 2k bp downstream of TFAP2A after querying the illumina 450K annotation file. TFAP2A was a key transcription factor in retinal development and could induce apoptosis in RB, suggesting TFAP2A was an ideal diagnostic biomarker for RB.

### Performance of RB methylation markers in RB tissue

**Figure 3:**
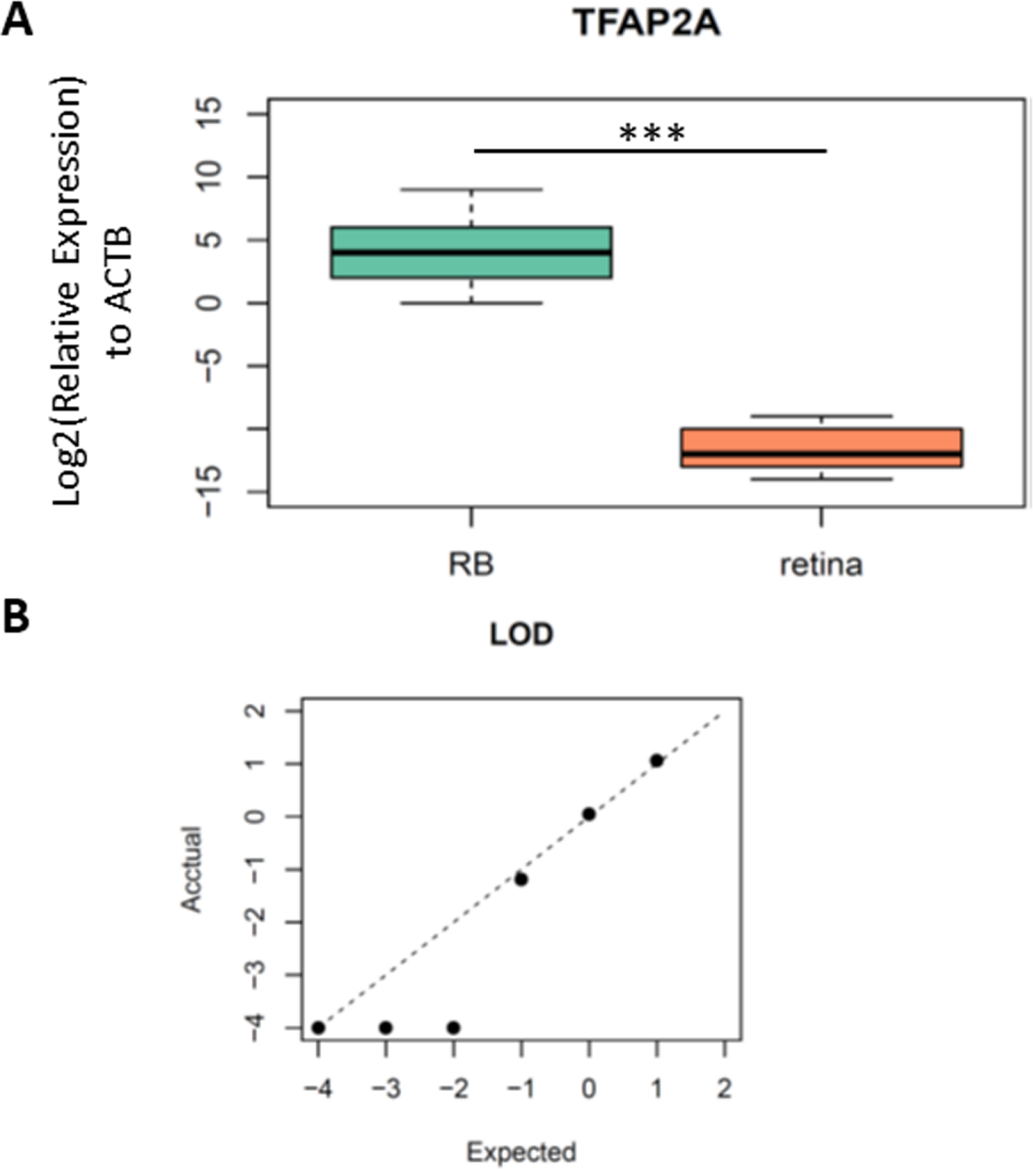
The performance of TFAP2A MSP assay in RB and normal retina tissue. (A) Boxplot showing the difference of relative expression of TFAP2A methylation between RB and retina. Student’s t test; ***, *p* < 0.001. (B) Dot plot showing the limit of detection (LOD) of our MSP assay. Axes scale, log10(ng/ul of ctDNA concentration).

As we identified TFAP2A was a potential diagnostic biomarker for RB, we then designed the primers and probe for the methylation specific PCR (MSP) in this region to validate clinical utility of TFAP2A for RB diagnosis. The forward and reverse primers were designed via MethPrimers (http://www.urogene.org/cgi-bin/methprimer2/MethPrimer.cgi). The sequences of primers and probes were listed in Table S1. To test the analytic validity of our primers and probe, we firstly performed a MSP on bisulfite-converted fully methylated or unmethylated standards. As we expected, only fully methylated standards formed PCR products. Then, we detected the methylation levels of TFAP2A in the tissue samples from five RBs and five non-RB retina to further confirm the hypermethylated TFAP2A is a diagnostic biomarker for RBs (Table 1). MSP results showed a significant difference of TFAP2A methylation level between RB and retina (Fig. 3A, Student’s t test, *p* = 8.107 × 10^−05^). To further confirm the analytic validation of our TFAP2A methylation assay, we detected the limit of detection (LOD) by serial dilution of RB tissue samples. The results of LOD demonstrated that our TFAP2A methylation assay could identify RB tissue from down to 1 ng DNA tissue sample. The correlation of actually detected methylated TFAP2A with the expected one also showed the linear pattern of our methylated TFAP2A assay (Fig. 3B). These results suggested the methylation status of TFAP2A was a promising diagnostic tool for RB.

### Performance of RB methylation markers in cfDNA

**Figure 4:**
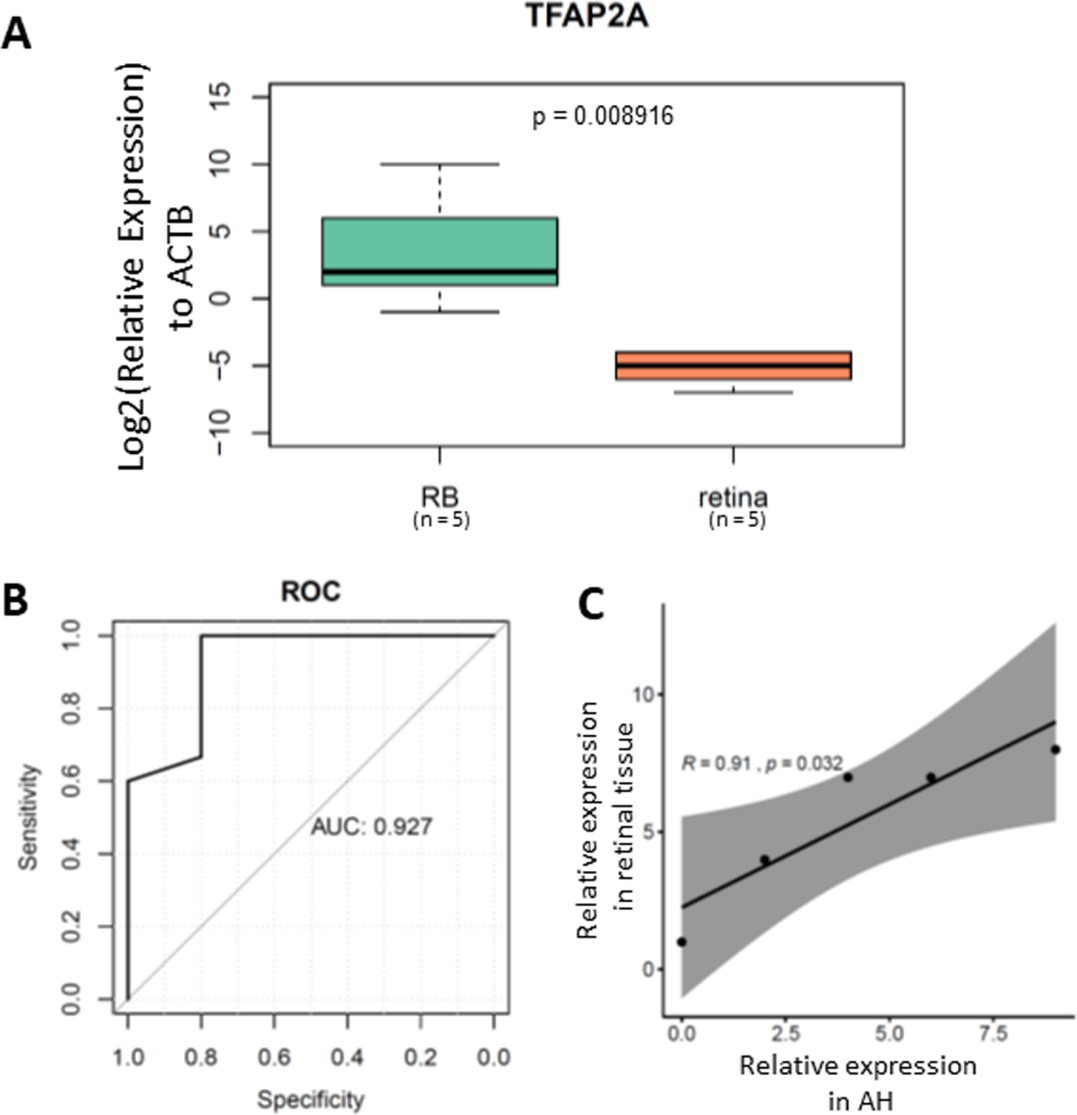
methylated TFAP2A is diagnostic biomarker for RB. (A) Boxplot showing the relative methylated TFAP2A. (B) ROC showing the TFAP2A methylation assay has a 92.7% discrimination diagnosis for RB in aqueous humor samples.

To further validate the clinical application of our TFAP2A methylation assay, we tested its performance in cfDNA isolated from RB aqueous humor. After cfDNA isolation from aqueous humor followed by bisulfite conversion, we performed the MSP on a total of 20 ctDNA samples from aqueous humor (Table 1). Our results demonstrated a significant methylated TFAP2A from RB cfDNA (Fig. 4A). The Area Under the Curve (AUC) of our methylated TFAP2A assay showed a 92.7% accuracy for diagnosing RB through ctDNA (Fig. 4B). We also correlated the methylated TFAP2A in AH with that in RB tissue. The Pearson correlation analysis suggested that the TFAP2A MSP result from AH was consistent with that from RB tissue (*R* = 0.91, *p* = 0.032, Fig. 4C). These results suggested our TFAP2A cfDNA methylation assay has the potent of diagnosis for RB.

## Discussion

Herein, we report a DNA methylation-based assay targeting TFAP2A used to detect RB from cfDNA of AH. We optimize this assay for detection of methylated TFAP2A as low as 100 pg input DNA. In this proof-of-concept study, we demonstrate methylated TFAP2A is detected more frequently in RB than non-RB tissue and this assay has a significant discrimination of RB from benign diseases.

Epigenetic biomarkers, especially aberrant DNA methylation have becoming emerging biomarkers in clinical practice such as early cancer diagnosis and therapeutic prediction (Ma et al., 2018; Morokoff et al., 2020). A panel of limited numbers of CpG loci is informative to predict the presence of tumor or not. In addition, a million of CpG loci sophisticatedly screened by a few machine learning approaches as a pan-cancer diagnostic tool can surrogate the approximate 30 million genome CpG loci confirm that DNA methylation alteration is one of the early events in cancer development (Taylor, 2020). Methylation-based assay is also widely used in clinical practice. In glioma, hypomethylated MGMT promoter indicates the additional survival benefit from alkylation agent temozolomide (Butler et al., 2020). Hence, a few commercial kits or assay targeting DNA methylation are available now for clinical usage. Although the NGS based approaches or kits can yield more comprehensive profiles of DNA methylation patterns in given samples, they are still not recommended for clinical practice yet due to the relatively higher costs as compared with PCR-based approaches. In RB, a hypermethylation pattern of RB1 gene and epigenetic silencing are observed when it progresses (Davalos-Salas et al., 2011; Anwar & Lehmann, 2018). We identified another hypermethylated CpG loci other than RB1, suggested there is some hypermethylated regions in RB as like as other types of cancer. These CpG loci are likely RB specific biomarkers and assays targeting these CpG loci allow to determine the likelihood of the presence of RB.

TFAP2A acts as a sequence-specific DNA-binding transcription factor that recognizes and binds to specific DNA sequences and recruits transcription mechanisms. Its binding site is a GC-rich sequence, which exists in the cis-regulatory region of several genes (Williams & Tjian, 1991). TFAP2A is a 52 kD retinoic acid inducible transcriptional regulator, which can bind to the consensus DNA binding sequence in the promoter of SV40 and metallothionein. TFAP2A is expressed in neural c cell lineage, and its highest level of expression corresponds to that of early neural c cells, which indicates that TFAP2A plays a role in its differentiation and development. In the process of neural tube closure in mice, the transcription factor TFAP2A is expressed in the ectoderm and neural cells removed from the cranial fold. Cranial nerve rest cells provide information on the pattern of craniofacial morphogenesis and produce most of the skull and cranial ganglia (Williams & Tjian, 1991; Mitchell, Wang & Tjian, 1987; Le Douarin, Ziller & Couly, 1993). TFAP2A knockout mice died of cranioabdominal schizophrenia during the perinatal period, with severe malformations of the face, skull, sensory organs and cranial ganglia (Schorle et al., 1996). Homozygous knockout mice also have neural tube defects, followed by craniofacial and body wall abnormalities (Zhang et al., 1996). Mutations in the TFAP2A gene usually lead to cleft lip in the middle eye and cause branch ocular facial syndrome (Dixon et al., 2011). In a family with branching eye syndrome (BOFS), a 3.2 Mb deletion was detected on chromosome 6 (Pelling et al., 1986). Sequencing of candidate genes in this region in 4 other unrelated BOFS patients showed that there were 4 different de novo missense mutations in exons 4 and 5 of the TFAP2A gene. Mutations in the TFAP2A gene can also cause branching eye and face syndrome, (Pelling et al., 1986) which has overlapping features with Van der Woude syndrome, such as orofacial cracks. These findings indicate that TFAP2A are critical in the developmental pathway, and a variant is identified in the regulatory region, which is essentially the cause of common complex diseases.

We also noticed there are some limitations in our study. Due to the limited sample size, we haven’t validated our test in a larger cohort, which is important before it is taken into clinical practice. Secondly, we also haven’t included other congenital retinal diseases in this study. For the future study, we will include other congenital retinal diseases to validate the specificity of our DNA methylation marker. Lastly, the biological mechanism of aberrant DNA methylation patterns including hypermethylated TFAP2A with RB1 mutation has yet been elucidated. We still do not know whether RB1 mutation is one of the contribution factors that resulting in the hypermethylated TFAP2A. A few experiments are required to explore the biological function of aberrant DNA methylation in RB.

## Conclusion

In summary, we firstly reported two RB specific hypermethylated CpG loci through bioinformatic analyses and an MSP targeting the region covering these two CpG loci has demonstrated a significant discrimination of RB sample from non-RB samples. The clinical validation of this assay is also validated in cfDNA from AH with high accuracy. Further evaluation of this assay across a broad range of clinical disorders is indicated.

##  Supplemental Information

10.7717/peerj.10830/supp-1 Supplemental Information 1 The sequences of primers and probe used for methylation specific PCR of TFAP2A

10.7717/peerj.10830/supp-2 Supplemental Information 2 Raw data

10.7717/peerj.10830/supp-3 Supplemental Information 3 REMARK checklist

## Additional Information and Declarations

### Data Availability

The following information was supplied regarding data availability: Code is available at Figshare: Wang, Sha (2020): 101DMP_normal.R. figshare. Software. https://doi.org/10.6084/m9.figshare.13082504.v1
Dataset is also available at Figshare: Wang, Sha (2020): DNAme_Retina.RData. figshare. Dataset. https://doi.org/10.6084/m9.figshare.13082486.v1.
