# Supplementary material for: The methylation level of TFAP2A is a potential diagnostic biomarker for retinoblastoma: an analytical validation study
Source: PeerJ. 2021 Mar 2;9:e10830. doi: 10.7717/peerj.10830 (PMC7934648; doi:10.7717/peerj.10830)
Supplement: Supplemental Information 1 [file peerj-09-10830-s001.docx]

| **Supplementary table 1. The sequences of primers and probe used for methylation specific PCR of TFAP2A** | | |
| --- | --- | --- |
|  |  | **sequences** |
| **TFAP2A** | **forward primer** | CGTAAGCGCGTTAAGGGAGAC |
|  | **reverse primer** | AAAAAATATAAACGACCTAAACAACG |
|  | **probe** | TTTGGACGCGAGTGTCGGTGGTCGTTGTTT |
